# Characteristics of adipocytokine expression by local fat depots of the heart: Relationship with the main risk factors for cardio-vascular diseases

**DOI:** 10.3389/fendo.2022.991902

**Published:** 2022-09-08

**Authors:** Olga V. Gruzdeva, Yulia A. Dyleva, Ekaterina V. Belik, Maxim Yu. Sinitsky, Kiril A. Kozyrin, Olga L. Barbarash

**Affiliations:** Federal State Budgetary Scientific Institution, Research Institute for Complex Issues of Cardiovascular Diseases, Kemerovo, Russia

**Keywords:** adiponectin, leptin, leptin receptor, IL-6, adipose tissue, risk factors cardiovascular diseases

## Abstract

In our study we investigated the relationships between adipocytokines in adipose tissue (AT) and cardiovascular disease (CVD) risk factors; (2) Methods: fat tissue biopsies were obtained from 134 patients with stable CAD undergoing coronary artery bypass grafting and 120 patients undergoing aortic or mitral valve replacement. Adipocytes were isolated from subcutaneous (SAT), epicardial (EAT), and perivascular AT (PVAT) samples, and cultured for 24 h, after which gene expression of adipocytokines in the culture medium was determined; (3) Results: men showed reduced ADIPOQ expression in EAT and PVAT, LEP expression in PVAT, and LEPR expression in SAT and PVAT compared to women. Men also exhibited higher SAT and lower PVAT IL6 than women. Meanwhile, dyslipidemia associated with decreased ADIPOQ expression in EAT and PVAT, LEPR in EAT, and IL6 in PVAT. Arterial hypertension (AH) associated with low EAT and PVAT ADIPOQ, and high EAT LEP, SAT, as well as PVAT LEPR, and IL6 in SAT and EAT. ADIPOQ expression decreased with increased AH duration over 20 years against an increased LEP background in ATs. Smoking increased ADIPOQ expression in all ATs and increased LEP in SAT and EAT, however, decreased LEPR in PVAT. Patients 51–59 years old exhibited the highest EAT and PVAT LEP, IL-6, and LEPR expression compared to other age groups; (4) Conclusions: decreased EAT ADIPOQ expression against an increased pro-inflammatory IL6 background may increase atherogenesis and contribute to CAD progression in combination with risk factors including male sex, dyslipidemia, and AH.

## Introduction

Morbidity and mortality from cardiovascular diseases (CVD) remain prevalent in many countries, despite ongoing prophylaxis and the introduction of new treatment methods (1). The epicardial adipose tissue (EAT) and perivascular (PVAT) are important in the pathogenesis of atherosclerosis as they are located in close proximity to the myo-cardium and coronary arteries and function as active endocrine organs, being able to synthesize and produce adipocytokines. In patients with elevated cardiovascular risk, higher pro-inflammatory adipocytokine levels are observed in EAT than subcutaneous adipose tissue (SAT) (2), and the EAT of patients with severe coronary artery disease (CAD) expresses less adiponectin (3). Moreover, EAT thickness correlates with metabolic risk factors and contributes to coronary artery atherosclerotic plaque development (4).

Adiponectin, the main protein secreted by adipocytes, exhibits cardioprotective, an-ti-diabetic, anti-atherogenic, and anti-inflammatory effects, unlike other adipokines (5). Low adiponectin levels are associated with arterial hypertension (AH), obesity, insulin resistance, type 2 diabetes mellitus, and myocardial infarction (MI) (6). Conversely, leptin has pro-inflammatory and prothrombotic effects (7), whereas pro-inflammatory inter-leukin 6 (IL-6) can elicit hypertrophy-inducing effects and is an independent predictor of CAD vessel disease (8). Recently, an increasing body of evidence has demonstrated that the expression of adipocytokines differ depending on the location of the fat depot.

Although assessing cardiovascular risk based on traditional risk factors has high prognostic value, identifying new parameters can significantly improve the stratification model for patients with cardiovascular disease. Various factors, including pro-inflammatory markers, that have demonstrated potential association with athero-genesis, are produced by AT (9), however, data regarding the association between age, sex, and other parameters, with adiponectin, leptin, and IL-6 levels in the various local fat depots, remain limited and are often contradictory. Accordingly, determining the factors that impact the course of CAD, as well as the associated prognosis, remains critical (10). Moreover, as atherosclerosis constitutes a multifactorial disease influenced by both un-modifiable (e.g., sex, age) and modifiable (smoking, dyslipidemia) factors, clarification of the pathogenetic relationships between adipocytokines and CVD risk factors is necessary. Toward this end, we evaluated the expression of adiponectin, leptin, its soluble receptor (sOB-R), and IL-6 in EAT, PVAT, SAT, and their relationships with the main CVD risk factors.

## Materials and methods

### Study design and patients

This study was performed at the Federal State Budgetary Institution’s Research In-stitute for Complex Issues of Cardiovascular Diseases. We examined 134 patients with a median age of 65.6 (49.3; 70.3) years with CAD who underwent elective coronary artery bypass grafting (CABG) and 120 patients median aged 60.47 (45.2; 63.2) years with aortic or mitral valve replacement. Exclusion criteria included: 1) > 75 years of age; 2) clinical conditions including MI, type 1 or type 2 diabetes mellitus, anemia, autoimmune diseases, liver or kidney failure, infectious or inflammatory diseases, and oncological diseases.

### Cardiovascular risk factor assessment

Traditional cardiovascular risk factors and patient treatment were recorded. AH was defined as systolic blood pressure > 140 mm Hg Art., diastolic blood pressure > 90 mm Hg. Dyslipidemia was defined as a previously detected increase in total serum cholesterol (> 200 mg/dl), triglycerides (>200 mg/dl), or low-density lipoprotein (LDL) cholesterol (> 150 mg/dl) for at least 1 year or use of lipid-lowering drugs. Smoking was classified as current or former smokers; current smoking status was defined as at least one cigarette daily over the last year.

### Sample collection and evaluation

SAT, EAT, and PVAT biopsies (3 to 5 g) were obtained during aortocoronary bypass surgery and aortic or mitral valve replacement. SAT samples were obtained from the subcutaneous tissue of the lower angle of the mediastinal wound. EAT was sourced from its largest source, from the right heart (right atrium and ventricle) and PVAT were ob-tained from the area of the right coronary artery. Adipocytes were isolated from adipose tissues under sterile conditions in a laminar flow hood (BOV-001-AMS MZMO, Millerovo, Russia), as previously described (11). Adipocytes were counted in a Goryaev chamber. Cell viability was evaluated according to the method described by Suga et al. (12). Adipocytes (20 × 10^5^) were seeded into a 24-well plate (Greiner Bio One International GmbH, Kremsmünster, Austria), and the volume in each well was adjusted to 1 mL with culture medium, as previously described (11). Cells were incubation for 24 h at 37 ± 1°C in an atmosphere of 5% CO2 and 10% oxygen. The adipocytes were then immediately processed for RNA extraction to determine adipocytokine gene expression.

### RNA extraction

Total RNA was isolated from adipocytes using the commercial RNeasy^®^ Plus Uni-versal Mini Kit (Qiagen, Hilden, Germany), according to the manufacturer’s instructions with slight modifications, as described previously (13). The quantity and quality of puri-fied RNA were assessed using a NanoDrop 2000 Spectrophotometer (Thermo Fisher Scientific) by measuring the light absorbance at 280 nm, 260 nm, and 230 nm and calcu-lating the 260/280 (A260/280) and 260/230 (A260/230) ratios. The integrity of the RNA was determined by electrophoresis in agarose gel, followed by visualization using the Gel Doc™ XR+ System (Bio-Rad, Hercules, CA, USA). Extracted RNA was stored at –70°C.

### cDNA synthesis

Single-stranded cDNA was synthesized using the High-Capacity cDNA Reverse Transcription Kit (Applied Biosystems, Foster City, CA, USA) on a VeritiTM 96-Well Thermal Cycler (Applied Biosystems). Reverse transcription was performed using the program suggested by the manufacturer. The quantity and quality of synthesized cDNA were assessed using a NanoDrop 2000 Spectrophotometer. Samples were stored at –20°C.

### Gene expression determination

Expression of adiponectin (ADIPOQ), leptin (LEP), soluble leptin receptor (LEPR) and IL6 genes was evaluated by quantitative real-time polymerase chain reaction (qPCR) using TaqManTM Gene Expression Assays (ADIPOQ Hs00605917_m1, LEP Hs00174877_m1, LEPR Hs00174497_m1, IL6 Hs00174131_m1, Applied Biosystems, USA) on a ViiA 7 Real-Time PCR System (Applied Biosystems). Each 20 µL reaction mix contained 10 µL of TaqManTM Gene Expression Master Mix (Applied Biosystems), 1 µL of TaqManTM Gene Expression Assay (Applied Biosystems), and 9 µL of cDNA template comprising 100 ng of cDNA + nuclease-free water). Samples were amplified under the following thermal cycling conditions: 2 min at 50°C, 10 min at 95°C, 40 cycles of 15 sec at 95°C and 1 min at 60°C. As a negative control, 20 µL of reaction mix with no cDNA template was used. For each sample and negative control, three technical replicates were prepared.

The results were normalized using reference genes HPRT1, GAPDH, and B2M. Test gene expression was calculated using the Pfaffl method and expressed on a logarithmic (log10) scale as a multiple change relative to the control samples (14).

### Statistical analysis

Statistical analysis was performed using GraphPad Prism 6 (La Jolla, CA, USA) and Statistica version 9.1 (Dell Software, Inc., Round Rock, TX, USA). The Kolmogorov–Smirnov test was used to verify normal distribution of data. For non-normally distributed variables, data were presented as median (Me) and 25th and 75th quartiles (Q1; Q3). Comparison of two independent groups was carried out using the nonparametric Mann-Whitney test. Differences between three groups were compared using one-way analysis of variance (ANOVA) for continuous variables. Categorical variables are ex-pressed as percentages and compared using chi-squared test or Fisher’s exact test. P values < 0.05 were considered statistically significant.

## Results

### Adipocytokine gene expression in the culture medium of adipocytes collected from the adipose tissue of patients with coronary artery disease and heart defects

Analysis of the clinical and anamnestic characteristics revealed that 75% of the sub-jects were men, AH was observed in 90.5% of all patients, angina pectoris in 97.63%, family history of CAD in 59.5%, previous MI in 67.86%, history of cerebrovascular acci-dent/transient ischemic attack in 7.14%, and 69.0% of patients smoked (**Table 1**). Patients received standard therapy with antiplatelet agents, beta-blockers, ACE inhibitors, and HMG-CoA reductase inhibitors.

**Table 1:** Clinical and medical history of patients with coronary heart disease.

Parameter	Patients with CAD,n=134	Patients with aortic or mitral valve replacement,n=120	Р
Males	80 (60)	58 (48.3)	0.053
Age, years	65.6 (49.3; 70.3)	60.47 (45.2; 63.2)	0.061
Body mass index, kg/m^2^	29.57 (25.19; 33.22)	26,59 (23.44;28.31)	0.075
Overweight	45 (33.58)	7 (5.8)	0.011
Arterial hypertension	75 (56)	28 (23.3)	0.014
Dyslipidemia	57 (42.5)	12 (10)	0.009
Smoking	64 (47.8)	14 (11.7)	0.017
Family history of coronary artery disease	79 (58.9)	42 (35)	0.049
Angina prior to MI	118 (88.1)	0	
Previous MI	91 (67.9)	0	
History of cerebrovascular accident/transient ischemic attack	10 (7.5)	0	
Atherosclerosis of other pools	21 (15.7)	0	
No angina	4 (3)	120 (100)	0.001
Angina I FC	0	0	
Angina II FC	62 (46.3)	0	
Angina III FC	68 (50.7)	0	
Angina IV FC	0	0	
CHF I FC	16 (11.9)	16 (13.3)	0.066
CHF II FC	11 (8.2)	54 (45)	0.003
CHF III FC	5 (3.7)	38 (31.6)	0.001
CHF IV FC	0	0	
Atherosclerosis of the 1st coronary artery	12 (9)	0	
Atherosclerosis of the 2st coronary artery	8 (6)	0	
Atherosclerosis of three or more coronary artery	114 (85.1)	0	
Ejection fraction, %	51.4 (43.7; 57.2)	53.1 (45.0;59.2)	0.048
C-reactive protein before surgery	2.79 (2.11;3.38)	3.02 (2.81;3.40)	0.302
**Treatment strategy/group of drugs**			
Aspirin	131 (97.8)	0	
Clopidogrel	21 (15.6)	0	
Warfarin	0	100 (83.3)	
β-blockers	131 (97.8)	109 (90.8)	0.177
Angiotensin-converting enzyme	101 (75.4)	91 (75.8)	0.525
Statins	134 (100)	89 (74.2)	0.061
Calcium channel blocker	103 (76.9)	86 (71.7)	0.143
Nitrates	11 (8.2)	10 (8.3)	0.673
Diuretics	102 (76.1)	118 (98.3)	0.055

Data are presented in n (%) or Me (Q1; Q3). CAD, coronary artery disease; Me, median; Q1, first quartile; Q3, last quartile; MI, myocardial infarction; CHF, chronic heart failure; FC, functional class.

Moreover, ADIPOQ expression in CAD patients was lower in EAT compared to SAT (p = 0.038) and PVAT (p = 0.027), while that of leptin was higher in EAT compared to SAT and PVAT (p = 0.003 and p = 0.002, respectively). Similarly, LEPR was more highly ex-pressed in EAT (p = 0.001) and PVAT (0.0003) compared to SAT, with that in the PVAT higher than in the EAT (p = 0.028). In addition, EAT was characterized by the highest IL-6 expression in comparison with the of SAT and PVAT samples (p = 0.001 and p = 0.025, respectively). Similar expression patterns were observed in the tissues collected from patients with heart defects. However, patients with defects were characterized by a higher ADIPOQ expression in EAT (p = 0.031), lower LEP expression in EAT (p = 0.004) and PVAT (p = 0.008), lower LEPR expression in the PVAT (p = 0.022), as well as lower IL-6 expression in EAT (p = 0.0002) and PVAT (p = 0.003) compared to patients with CAD.

### Adipocytokine gene expression in the adipocytes collected from adipose tissue based on the sex of coronary artery disease patients

**Figure 1:**
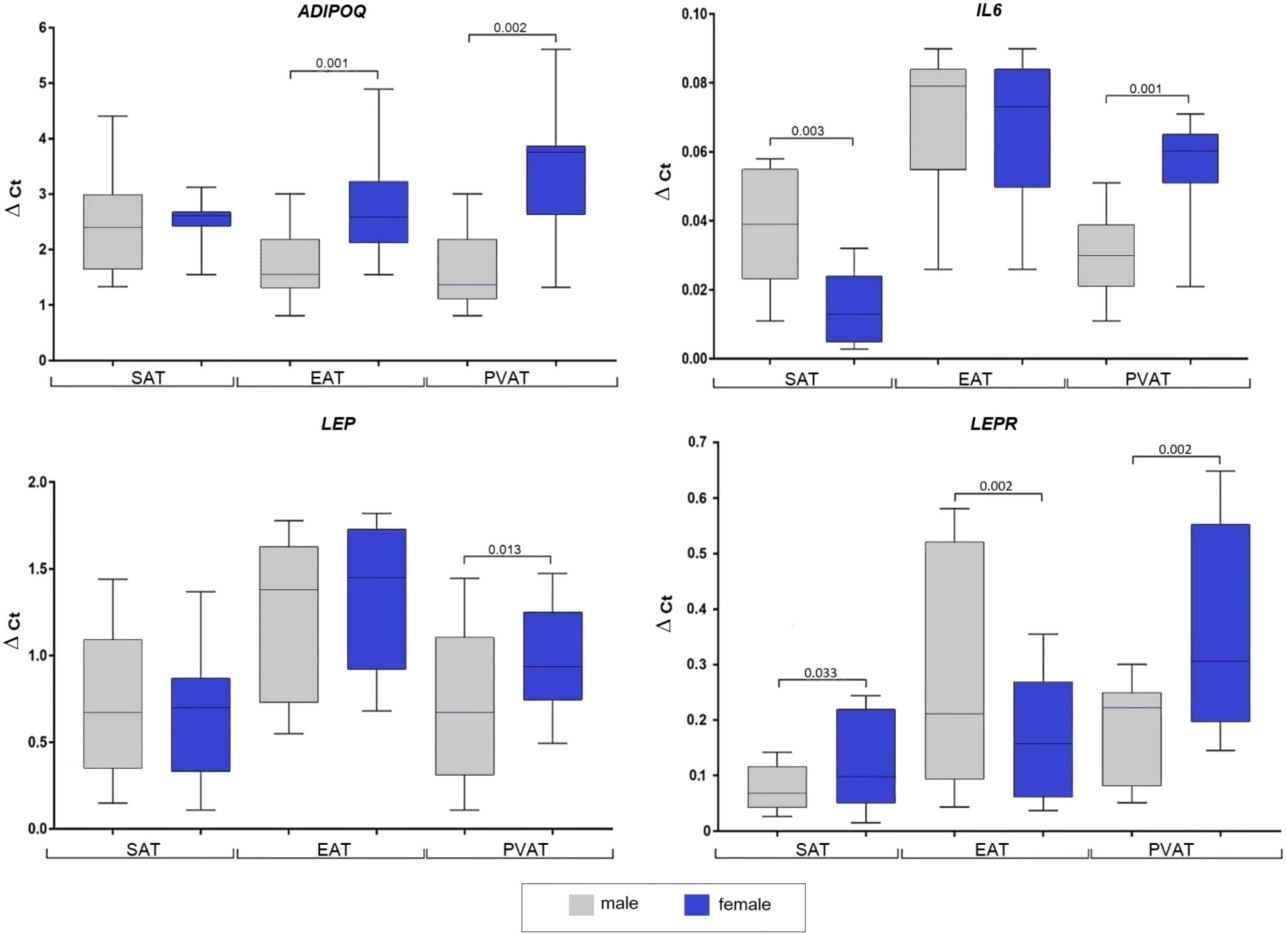
Adipocytokine genes expression in the subcutaneous, epicardial, and perivascular adipocytes based on the sex of patients with coronary artery disease. Data are presented in Me (Q1; Q3). SAT, subcutaneous adipose tissue; EAT, epicardial adipose tissue; PVAT, perivascular adipose tissue; p, level of statistical significance; sOB-R, soluble leptin receptor; IL6, interleukin 6.

Women exhibited higher ADIPOQ expression in EAT (2.5-fold, p = 0.001) and PVAT (2.8-fold, p = 0.002) compared to men, whereas expression in SAT did not differ between sexes (**Figure 1**).

Women also showed higher LEP mRNA expression in PVAT (1.4-fold, p = 0.013), while that in SAT and EAT did not significantly differ. In men, LEPR expression was lower in SAT (1.5-fold, p = 0.033) and PVAT (1.3-fold, p = 0.042), but higher in EAT (1.3-fold, p = 0.013). Also, the IL6 mRNA expression in men was significantly higher in SAT (3-fold, p = 0.003), however, was 2-fold lower in PVAT (p = 0.01), whereas no dif-ference was observed between sexes in EAT.

### Adipocytokine gene expression in the adipocytes collected from adipose tissue based on the age of coronary artery patients

**Figure 2:**
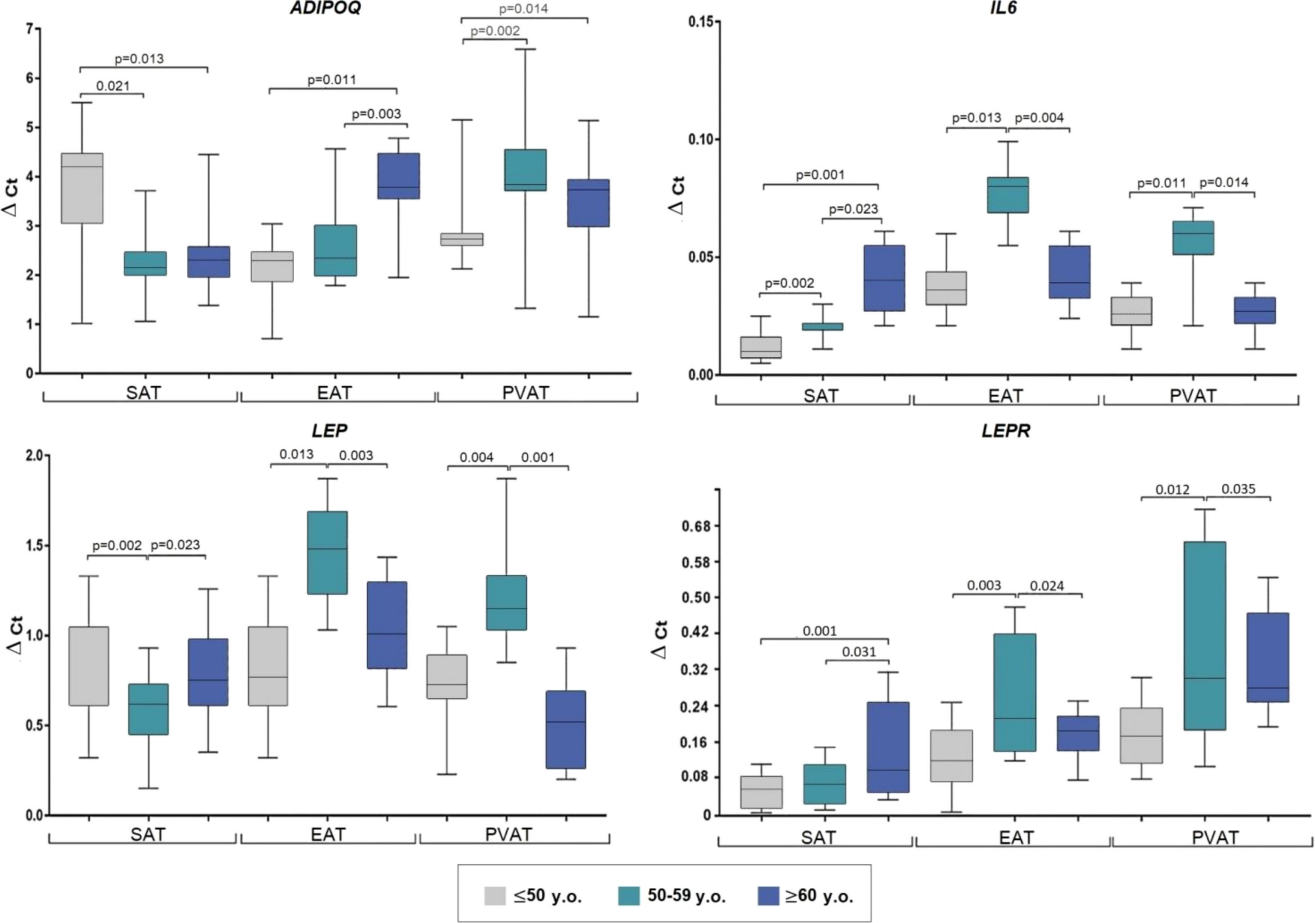
Adipocytokine gene expression in the adipocytes collected from adipose tissue based on the age of coronary artery patients. Data are presented in Me (Q1; Q3). SAT, subcutaneous adipose tissue; EAT, epicardial adipose tissue; PVAT, perivascular adipose tissue; p, level of statistical significance; sOB-R, soluble leptin receptor; IL6, interleukin 6.

Evaluation according to patient age (≤ 50 years; younger, 51–59; mid-age, and ≥ 60; older) revealed maximum SAT ADIPOQ expression in the younger group, which was equivalent to twice that observed in the mid- age (p = 0.021) and older (p = 0.013) groups (**Figure 2**).

In EAT, the highest ADIPOQ mRNA expression was observed in the older group, which was 1.7-fold that in the younger (p = 0.011) and mid-age (p = 0.003) groups. Lowest PVAT ADIPOQ expression was observed in the younger group which was approximately 1.4-fold lower than that in the mid-age (p = 0.002) and older (p = 0.014) groups.

The mid-age group exhibited decreased LEP in SAT (1.2-fold) compared to the younger (p = 0.002) and older (p = 0.023) groups, however, increased LEP levels were observed in EAT and PVAT of mid-age patients compared to the younger (2-fold, p = 0.013; 1.6-fold, p = 0.004, respectively) and older (1.5-fold, p = 0.003; 2.2-fold, p = 0.001, respectively) groups.

The older group exhibited the highest LEPR expression in SAT (3-fold, p = 0.001 vs. younger; 2.3-fold, p = 0.031 vs mid-age), whereas the mid-age group showed highest ex-pression in EAT and PVAT (1.6-fold, p = 0.003 and 1.8-fold, p = 0.012 vs younger; 1.1-fold, p = 0.024 and 1.2-fold, p = 0.035 vs older).

SAT IL-6 expression was higher in the older group (4-fold, p = 0.011 vs younger; 2-fold, p = 0.023 vs mid-age). Meanwhile, the mid-age individuals exhibited the highest IL6 expression in EAT (2.2-fold, p = 0.013 vs younger; 2.05-fold, p = 0.004 vs older) and PVAT (2.3-fold, p = 0. 011; 2.2-fold, p = 0.014 times, respectively) (**Figure 2**).

### Adipocytokine gene expression in the adipocytes collected from adipose tissue based on the presence of dyslipidemia in coronary artery disease patients

**Figure 3:**
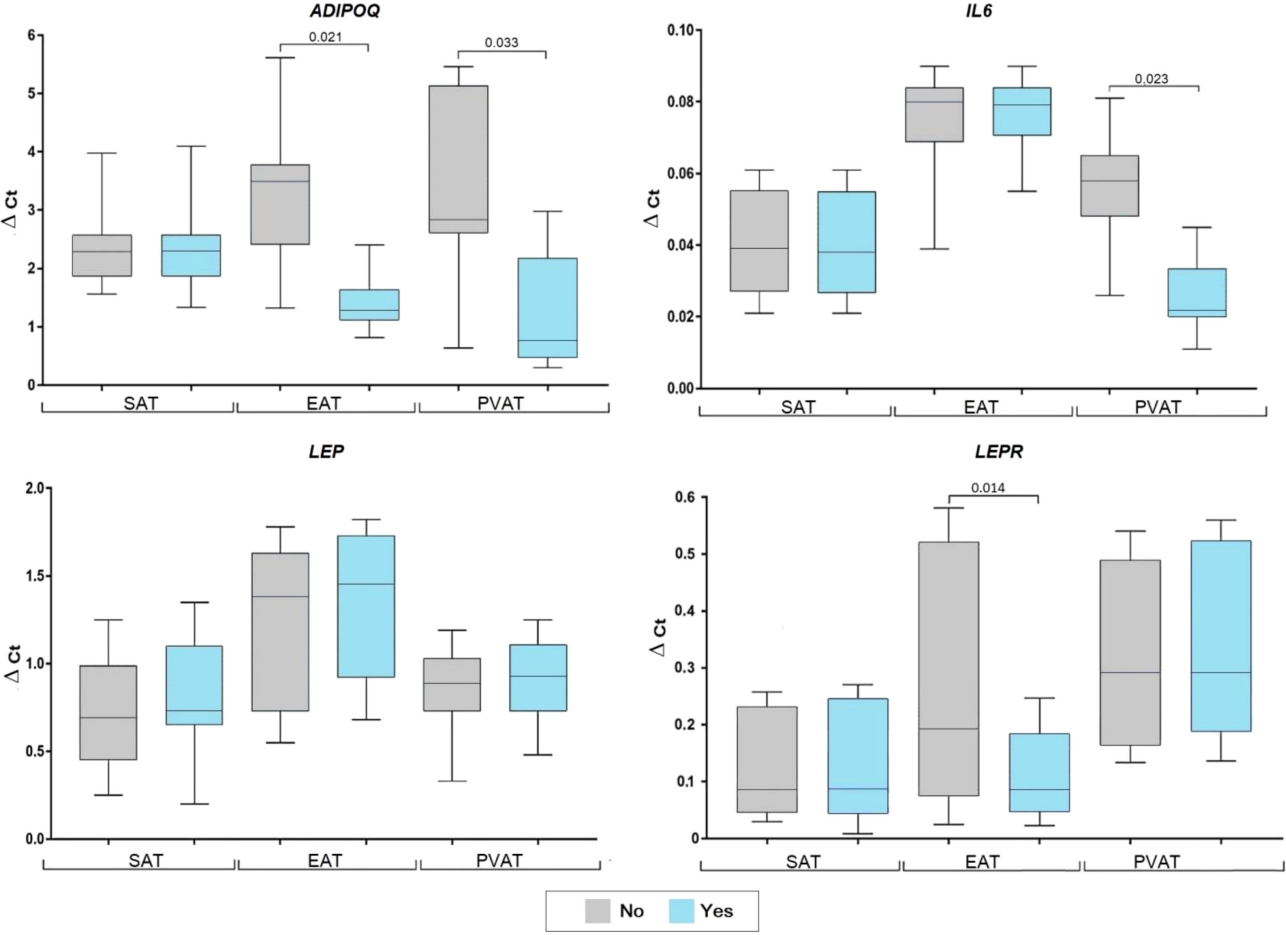
Adipocytokine gene expression in the adipocytes collected from adipose tissue based on the presence of dyslipidemia in coronary artery disease patients. Data are presented in Me (Q1; Q3). SAT, subcutaneous adipose tissue; EAT, epicardial adipose tissue; PVAT, perivascular adipose tissue; p, level of sta-tistical significance; sOB-R, soluble leptin receptor; IL6, interleukin 6.

In patients with dyslipidemia, ADIPOQ mRNA was decreased in EAT (2.7-fold, p = 0.021) and PVAT (3.6-fold, p = 0.033), however, did not differ in SAT (**Figure 3**).

Meanwhile, LEP expression was not correlated with dyslipidemia. Conversely, dyslipidemia was associated with a 2.1-fold (p = 0.014) decrease in LEPR mRNA in EAT and 2.3-fold decrease in IL6 in PVAT (p = 0.023). Alternatively, IL-6 did not differ based on dyslipidemia status in SAT and EAT.

### Adipocytokine gene expression in the adipocytes collected from adipose tissue depending based on arterial hypertension in coronary artery disease patients

**Figure 4:**
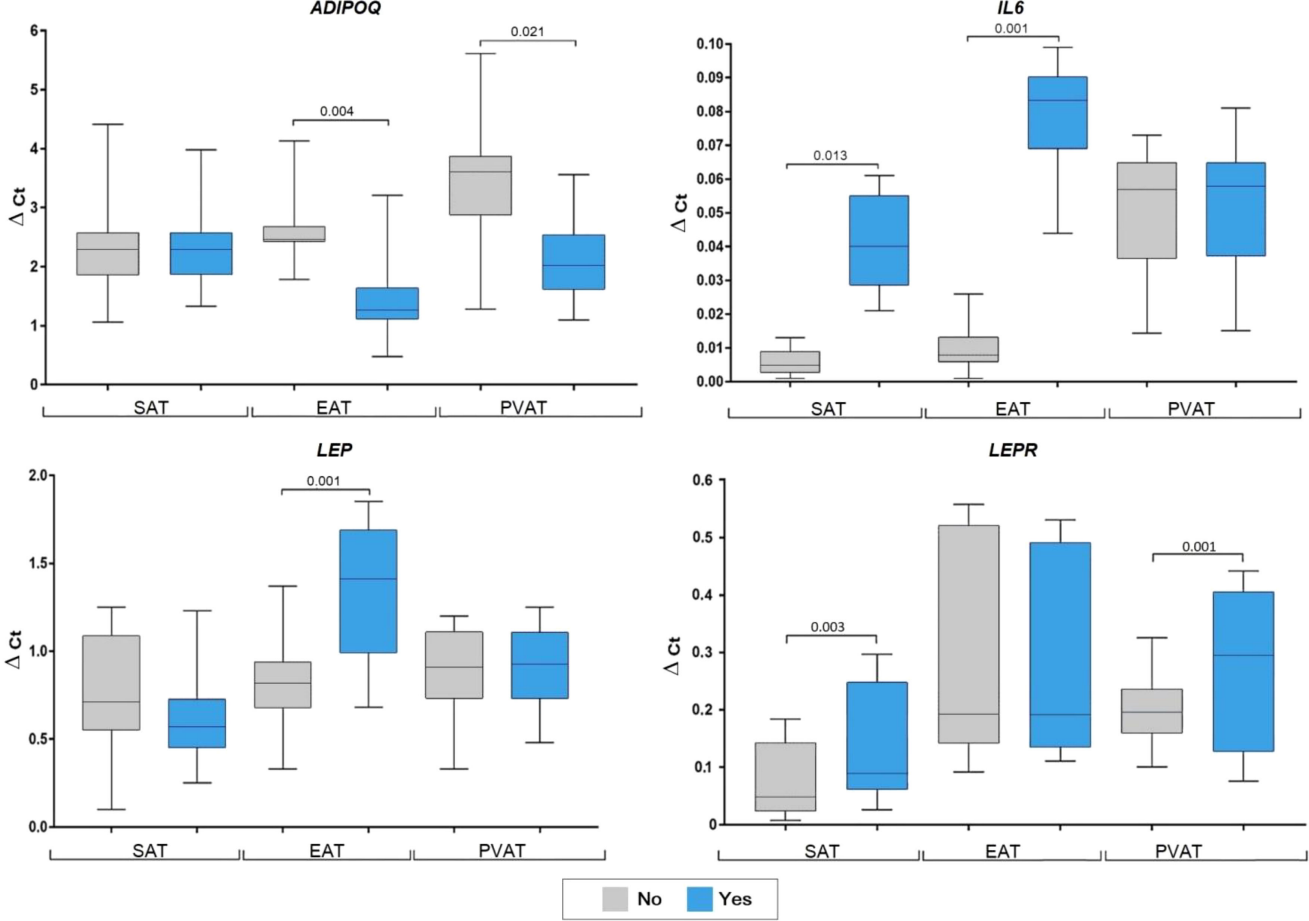
Adipocytokine gene expression in the adipocytes collected from adipose tissue based on arterial hypertension in coronary artery disease patients. Data are presented in Me (Q1; Q3). SAT, subcutaneous adipose tissue; EAT, epicardial adipose tissue; PVAT, perivascular adipose tissue; AH, arterial hypertension; p; level of statistical significance; sOB-R, soluble leptin receptor; IL6, in-terleukin 6.

AH in patients with CAD exhibited decreased ADIPOQ expression in EАT (2-fold, p = 0.004) and PVAT (1.8-fold, p = 0.021) coordinated with increased LEP mRNA expression in EАT (1.7-fold, p = 0.001), LEPR in SAT (3-fold, p = 0.003), and PVAT (1.7-fold, p = 0.001), but not in EAT. AH was also found to correlate with increased IL6 expression in SAT (8-fold, p = 0.013) and EAT (10.4-fold, p = 0.001) (**Figure 4**).

**Figure 5:**
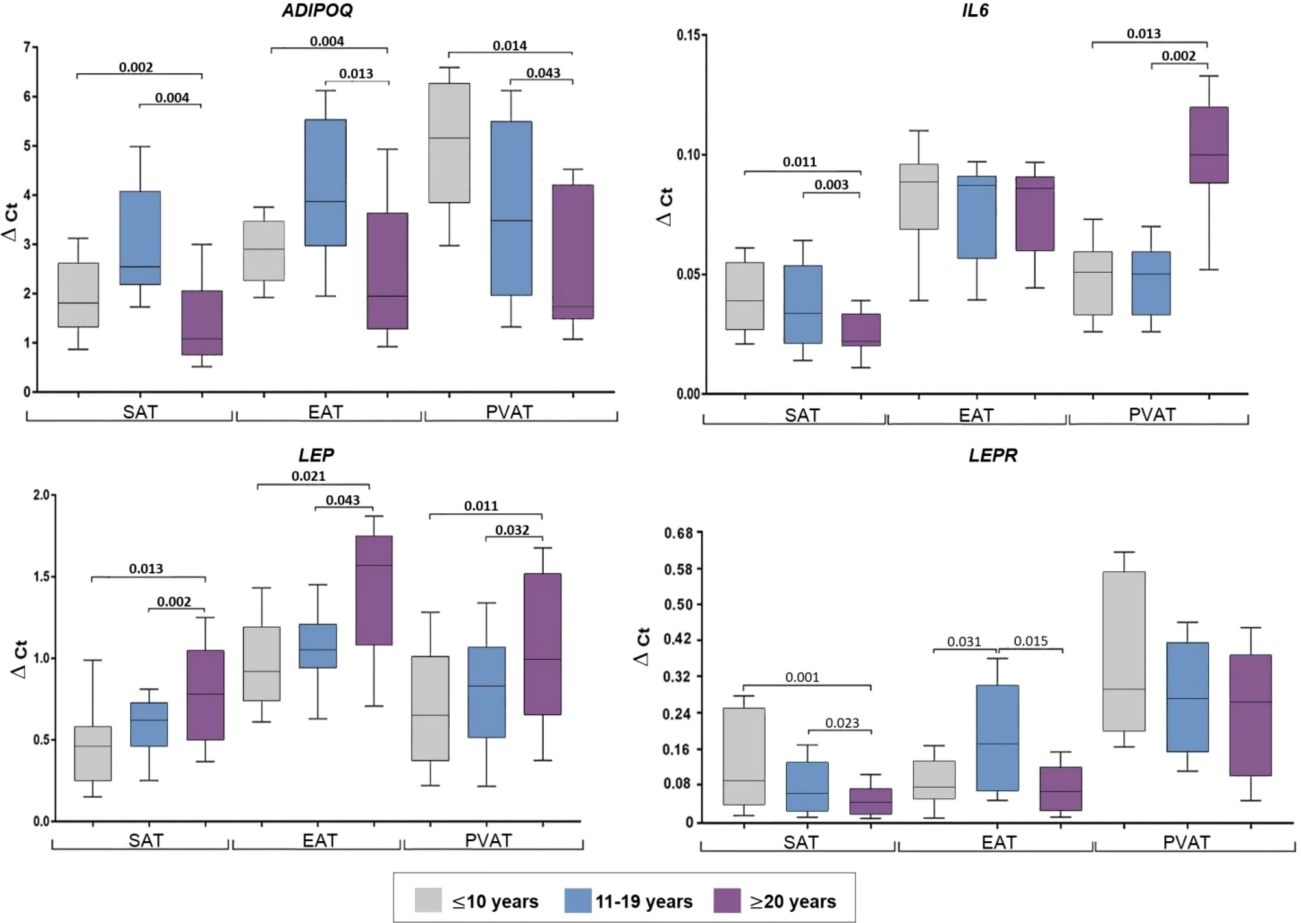
Adipocytokine genes expression in the subcutaneous, epicardial, and perivascular adipocytes based on duration of arterial hypertension in patients with coronary artery disease. Data are presented in Me (Q1; Q3). SAT, subcutaneous adipose tissue; EAT, epicardial adipose tissue; PVAT, perivascular adipose tissue; p, level of sta-tistical significance; sOB-R, soluble leptin receptor; IL6, interleukin 6.

AH duration also proved important for adipocytokine expression dynamics. Spe-cifically, in patients with AH for less than 10 years, LEPR was increased in SAT, whereas it was increased in the EAT of patients with AH for 11–19 years. Meanwhile, an AH du-ration of more than 20 years was associated with decreased ADIPOQ expression owing to LEP increases in all ATs, along with decreased IL6 in SAT, which was increased in PVAT (**Figure 5**).

### Adipocytokine gene expression in the adipocytes collected from adipose tissue based on smoking status in coronary artery disease patients

**Figure 6:**
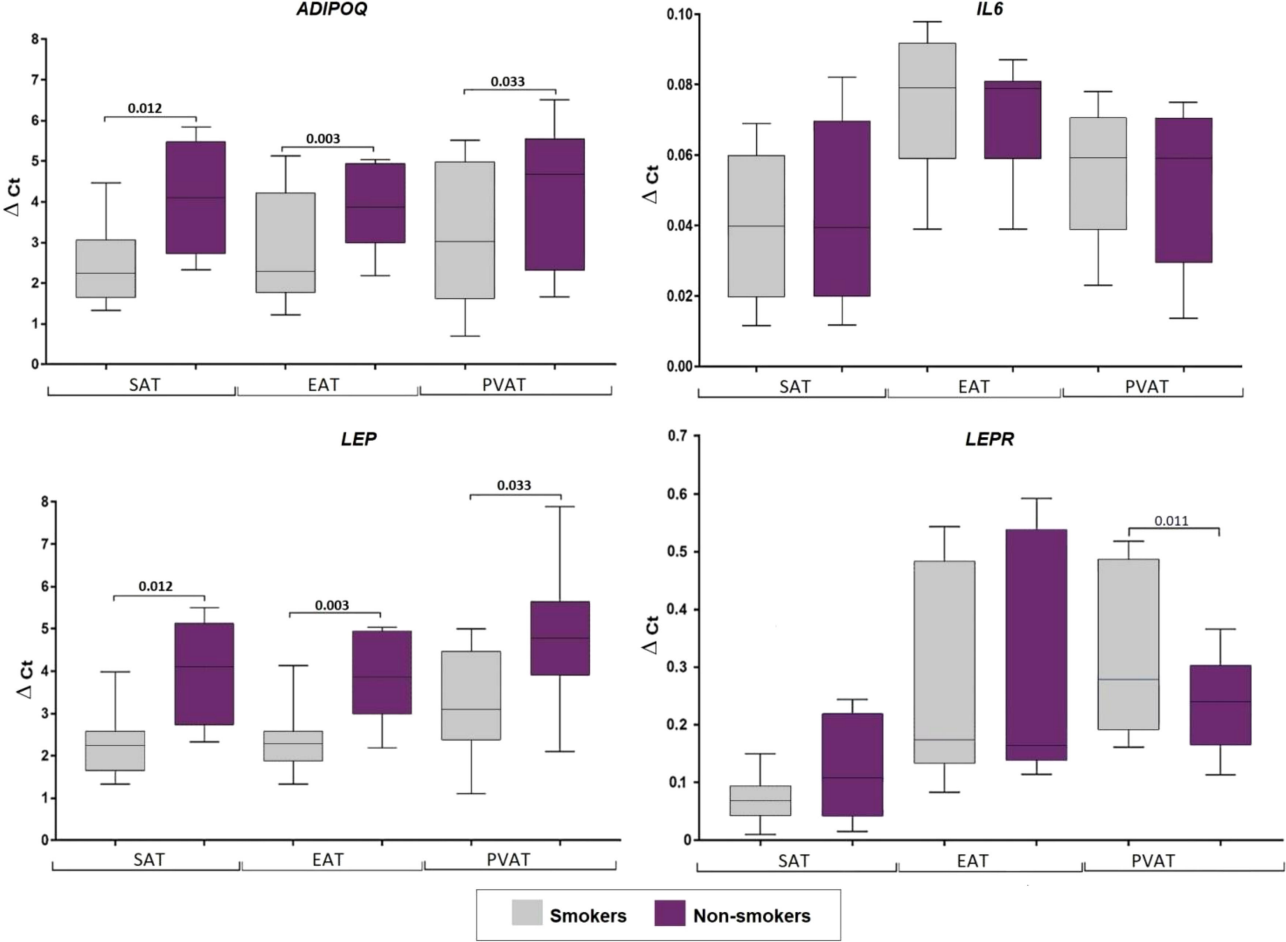
Adipocytokine gene expression in the culture medium of adipocytes collected from adipose tissue based on smoking status in coronary artery disease patients. Data are presented in Me (Q1; Q3). SAT, subcutaneous adi-pose tissue; EAT, epicardial adipose tissue; PVAT, perivascular adipose tissue; p, level of statistical significance; sOB-R, soluble leptin receptor; IL6, interleukin 6.

Smokers with CAD showed increased ADIPOQ expression in SAT (1.9-fold, p = 0.012), EAT (1.7-fold, p = 0.003), and PVAT (1.5-fold, p = 0.033), as well as increased LEP expression in SAT (1.6-fold, p = 0.024) and EAT (1.8-fold, p = 0.003). However, LEPR ex-pression was decreased (1.3- fold, p = 0.001) only in PVAT of smokers. No associations were found for smokers with CAD and IL6 expression (**Figure 6**).

## Discussion

### Sex-based differences

The results of this study revealed sexual dimorphism in the expression of adipo-cytokines in local fat depots, with primary EAT and PVAT localization. Specifically, men with CAD had lower ADIPOQ expression in EAT and PVAT, and lower LEP expression in PVAT compared to women. These results agreed with previously obtained data on lower LEP expression in the SAT of men relative to women (15). Our results also show a decrease in ADIPOQ and LEP expression in the EAT of male patients with CAD, while no difference was observed in the SAT (16). However, currently, a unanimous opinion has not been reached regarding the relationship between sex and the level of ADIPOQ in the PVAT. Thus, some researchers believe that sex affects both ADIPOQ expression and secretion, while others note differences only in the level of adiponectin secretion (17).

The revealed differences in adipokine expression may be due to the influence of sex hormones. For example, androgens, including testosterone, can cause dysfunction of AT through repression of ADIPOQ and LEP mRNA transcription *via* blocking of RNA polymerase and formation of a transcriptional complex (18). Moreover, Machinal-Quélin, F. at al. investigated the effects of *in vitro* 24-hour exposure to androgens and estrogens on LEP expression in the SAT of men and women. In men, only high concentration di-hydrotestosterone (100 nM) caused a decrease in the level of LEP expression, while in women 17-estradiol (10–100 nM) increased the expression of LEP. The authors suggested that the sexual dimorphism of LEP expression in humans is due to estrogen recep-tor-dependent stimulation of LEP expression in the AT by estrogens and estrogen pre-cursors in women (19). Further, a portion of testosterone becomes converted to estrogen through aromatization. McTernan P.G. at al. showed that the level of LEP expression in the adipocytes of women did not change in the presence of testosterone due to the low expression of aromatase in human adipocytes compared to pre-adipocytes (20).

When determining the level of IL6 expression, taking into account patient sex, men were found to have increased IL6 in SAT and decreased expression in PVAT, whereas no differences were observed in the EAT, compared to women. These results are consistent with our previous study, which demonstrated an increase of IL-6 expression in SAT during cardiac surgery (21).

### Age

It was hypothesized that the expression of adipocytokines changes with age, which is inextricably linked to CVD risk, an increase in the number and degree of coronary arteries, and the incidence of CAD. Our results demonstrate a clear association between age and adipocytokine mRNA levels. For instance, patients aged 50–59 were characterized by a low level of ADIPOQ in EAT, as well as high levels of LEP and IL-6 in EAT and PVAT. These expression patterns agree with the generally accepted opinion regarding increased pro-inflammatory activity in AT with age and, thus highlights the vulnerability of this patient population. The increase in IL-6 expression is likely caused by AT aging, which represents the main source of this cytokine. This was demonstrated in an *in vitro* study that treated visceral AT of C57BL/6 mice with lipopolysaccharides and found that IL-6 production was significantly higher in adipocyte cultures of mice aged 24 months com-pared to young mice (4 months). The authors also showed that IL-6 overproduction is regulated by the autocrine/paracrine action of IL-1β, which initiates inflammatory pro-cesses in old age (22).

### Dyslipidemia

Age-related AT dysfunction is believed to be associated with dyslipidemia, metabolic dysfunction, and mild chronic systemic inflammation, which affect the quality and duration of life (23). In the current study, the presence of dyslipidemia in CAD patients correlated with decreased ADIPOQ expression in EАT and PVАT. Similarly, EAT LEPR expression was lower in individuals with dyslipidemia, as was PVAT IL6 expression.

However, it was previously shown that ADIPOQ expression increases in the PVAT of men with CAD and a BMI above 30 kg/m2 compared to patients with a lower BMI (24), which, according to the authors, is reflective of the “obesity paradox.” Adiponectin affects the accumulation of LDL-C in the vascular wall, inhibiting its oxidation, as well as the transformation of macrophages into foam cells, and proliferation of smooth muscle cell neointima, while stimulating expression of the cholesterol ABCA1 ATP-binding transporter in the liver, thereby enhancing the biogenesis and reverse transport of HDL cholesterol exhibiting antiatherogenic properties (25). The observed decrease in ADIPOQ expression within patients with dyslipidemia, therefore, indicates negation of the above protective effects and contributes to the progression of atherosclerosis and vascular damage.

Furthermore, the decreased IL6 expression observed in the PVAT of CAD patients may result in increased accumulation of lipids in adipocytes, causing their hypertrophy. IL-6 is known to inhibit the expression of lipoprotein lipase (LPL), the most abundant of which is the cells of AT, heart and skeletal muscles. Normally, LPL is exported from ad-ipocytes to the endothelial lining of AT capillaries, where it cleaves the triglycerides of chylomicrons and VLDL, thereby regulating the concentration of triglycerides (26).

### Arterial hypertension

Analysis of adipocytokine expression based on the presence of AH demonstrated a decrease in ADIPOQ expression in EAT against the background of increased LEP and IL-6 expression. Moreover, the presence of hypertension for more than 20 years was found to be associated with a decrease in the level of ADIPOQ and increase in LEP within all AT types. Our results are consistent with those of a previous study that reported reduced expression of ADIPOQ and its receptors (AdipoR1 and AdipoR2) within the perivascular adipocytes of mice with angiotensin II-induced hypertension (27). Similarly, Teijeira-Fernandez et al. reported a decrease in ADIPOQ expression in the EAT of AH patients. The authors concluded that of ADIPOQ expression in EAT may be associated with AH status regardless of CAD or other concomitant diseases, which confirms the hypothesis regarding the effect of EAT on CVD (28).

In hypertension, the expression of LEP increases in EAT, which, given the possible proliferative effect of leptin and the effect on vascular permeability, may contribute to the progression of this disease. Research by Nepomuceno at al. demonstrated the presence of a direct correlation between LEP expression and blood pressure (29). The simultaneous decrease of ADIPOQ expression in the EAT and PVAT may also have an unfavorable affect as this adipokine attenuates vascular damage in hypertension.

### Smoking

When determining if smoking affects the expression of adipocytokines in CAD pa-tients, it was found that smoking is associated with an increase in ADIPOQ (in all types of AT), and LEP expression (in SAT and EAT), however, does not impact IL-6 expression. Similarly, a previous study sought to examine the effect of tobacco smoke *in vitro* and *in vivo* on the intracellular and extracellular distribution of adiponectin and its high mo-lecular weight form. Results showed that the total secretion of adiponectin was sup-pressed, while administration of tobacco smoke extract to mice reduced the adiponectin concentration in culture medium and the plasma of wild-type mice against the back-ground of its intracellular accumulation in cultured adipocytes. They further reported an enhancement of the adiponectin-retaining chaperone ERp44, localized in the endoplasmic reticulum, as well as suppression of the adiponectin secretion factor DsbA-L, following exposure to tobacco smoke. These results can help explain hypoadiponectinemia and the increased risk of developing T2DM in smokers due to its intracellular delay in the AT when exposed to tobacco smoke (30).

Moreover, the observed results regarding LEP expression agree with those of a pre-vious study that reported the effect of nicotine on the expression and secretion of leptin *in vitro*. They found that LEP expression did not differ significantly during the first 6 h of incubation with nicotine in cultured 3T3-L1 mouse adipocytes and AT explants from healthy women who underwent mammoplasty surgery. Meanwhile, LEP expression in 3T3-L1 mouse cells increased in the first hour and subsequently decreased by 45% after a 6-hour incubation with 0.5 μg/ml nicotine. However, low dose nicotine (0.05 μg/ml) did not affect LEP expression in 3T3-L1 cells. The observed change in LEP expression in cul-tured cells incubated with nicotine, and subsequent sharp decrease in plasma leptin concentration when smoking cigarettes suggested that the decrease in plasma leptin concentration in smokers is not associated with the direct effect of nicotine on LEP ex-pression and secretion, but rather with indirect exposure to catecholamines (31).

## Study limitations

Certain limitations were noted in this study. First, it was a single-centered study and, second, the sample size was small.

## Data availability statement

Data cannot be provided upon request due to the fact that the Local Ethics Committee cannot approve the transfer of any data on the patients participating in scientific and clinical researches which was conducted on the basis of the NII KPSSZ, since this is contrary to ethical standards and Federal Law of the Russian Federation “On Personal Data” No. 152-FZ of July 27, 2006.

## Ethics statement

The study protocol was approved by the institutional Local Ethics Committee, and was performed in ac-cordance with the World Medical Association’s Declaration of Helsinki on Ethical Principles for Medical Research Involving Human Subjects, 2000 edition, and the “GCP Principles in the Russian Federation”, approved by the Russian Ministry of Health. The patients/participants provided their written informed consent to participate in this study.

## Author contributions

Conceptualization, OG; data curation, OG, formal analysis YD, EB; investigation YD, EB, MS; methodology, OG, YD, administration, OB; resources, KK; supervision, OB; validation, OG; writing—original draft preparation, OG, YD, EB; writ-ing—review and editing, OG, YD. All authors contributed to the article and approved the submitted version.

## Funding

This research is conducted within the grant of the Russian Science Foundation project No. 22-15-20007 “Ceramide profile of local heart fat depots: clinical and pathogenetic significance and therapeutic potential”.

## Conflict of interest

The authors declare that the research was conducted in the absence of any commercial or financial relationships that could be construed as a potential conflict of interest.

## Publisher’s note

All claims expressed in this article are solely those of the authors and do not necessarily represent those of their affiliated organizations, or those of the publisher, the editors and the reviewers. Any product that may be evaluated in this article, or claim that may be made by its manufacturer, is not guaranteed or endorsed by the publisher.
